# Longitudinal Overlap and Metabolite Analysis in Spectroscopic MRI-Guided Proton Beam Therapy in Pediatric High-Grade Glioma

**DOI:** 10.3390/tomography11060071

**Published:** 2025-06-19

**Authors:** Abinand C. Rejimon, Anuradha G. Trivedi, Vicki Huang, Karthik K. Ramesh, Natia Esiashvilli, Eduard Schreibmann, Hyunsuk Shim, Kartik Reddy, Bree R. Eaton

**Affiliations:** 1 Department of Radiation Oncology, School of Medicine, Emory University, Atlanta, GA 30322, USA; abinand.rejimon@emory.edu (A.C.R.); anuradha.trivedi@emory.edu (A.G.T.); vicki.huang@emory.edu (V.H.); karthik.ramesh@emory.edu (K.K.R.); nesiash@emory.edu (N.E.); eschre2@emory.edu (E.S.); hshim@emory.edu (H.S.); 2 Department of Biomedical Engineering, Georgia Institute of Technology, Emory University, Atlanta, GA 30332, USA; 3 Winship Cancer Institute, School of Medicine, Emory University, Atlanta, GA 30322, USA; 4 Department of Radiology and Imaging Sciences, School of Medicine, Emory University, Atlanta, GA 30322, USA; kartik.mahasamudram.reddy@emory.edu; 5 Department of Radiology, Children’s Healthcare of Atlanta, Atlanta, GA 30342, USA

**Keywords:** spectroscopic MRI, pediatric high-grade glioma, proton beam therapy, longitudinal imaging analysis, magnetic resonance spectroscopy

## Abstract

Background: Pediatric high-grade glioma (pHGG) is a highly aggressive cancer with unique biology distinct from adult high-grade glioma, limiting the effectiveness of standard treatment protocols derived from adult research. Objective: The purpose of this report is to present preliminary results from an ongoing pilot study integrating spectroscopic magnetic resonance imaging (sMRI) to guide proton beam therapy and longitudinal imaging analysis in pediatric patients with high-grade glioma (pHGG). Methods: Thirteen pediatric patients under 21 years old with supratentorial WHO grade III-IV glioma underwent baseline and serial whole-brain spectroscopic MRI alongside standard structural MRIs. Radiation targets were defined using T1-weighted contrast enhanced, T2-FLAIR, and Cho/NAA ≥ 2X maps. Longitudinal analyses included voxel-level metabolic change maps and spatial overlap metrics comparing pre-proton therapy and post-. Results: Six patients had sufficient longitudinal data; five received sMRI-guided PBT. Significant positive correlation (R^2^ = 0.89, *p* < 0.0001) was observed between T2-FLAIR and Cho/NAA ≥ 2X volumes. Voxel-level difference maps of Cho/NAA and Choline revealed dynamic metabolic changes across follow-up scans. Analyzing Cho/NAA and Cho changes over time allowed differentiation between true progression and pseudoprogression, which conventional MRI alone struggles to achieve. Conclusions: Longitudinal sMRI enhanced metabolic tracking in pHGG, detects early tumor changes, and refines RT targeting beyond structural imaging. This first in-kind study highlights the potential of sMRI biomarkers in tracking treatment effects and emphasizes the complementary roles of metabolic and radiographic metrics in evaluating therapy response in pHGG.

## 1. Introduction

Pediatric high-grade glioma (pHGG) is an aggressive disease with a 3-year overall survival rate of less than 30% [1,2]. The distinct phenotypes of pHGGs compared to adult high-grade glioma (HGG) hamper the utility of current standard-of-care treatment pathways which are based upon adult HGG research [3,4,5]. pHGG treatment utilizes surgery, radiation therapy (RT), and varying systemic therapies according to molecular findings, though the prognosis remains dismal [6].

Proton beam therapy (PBT) offers significant dosimetric advantages over photon RT [7,8,9,10]. PBT delivers energy with high precision and minimizes damage to healthy tissues, making it ideal for pediatric, brain, and head and neck tumors [11,12,13,14,15,16]. Photon therapy, though more widely accessible and effective for many cancers, is less precise and leads to greater radiation exposure to the developing brain [17,18,19,20,21]. PBT is more expensive and is less studied due to its novelty, while photon therapy is affordable and supported by extensive clinical evidence. Current evidence suggests that PBT and photon-based therapies offer comparable overall survival (OS) rates for many cancer types, including lung, prostate, and head and neck cancers, as well as multiple pediatric brain tumors such as medulloblastoma, ependymoma, and low-grade glioma [22,23,24,25,26]. For cancers like esophageal cancer, some meta-analyses indicate that PBT may result in slightly improved OS compared to photon therapy, but these findings are not universal and are more pronounced in specific treatment scenarios [24]. PBT’s benefits are often associated more with minimizing side effects rather than improving survival, making it particularly valuable for pediatric patients and tumors located near critical structures like the brain or heart [27]. Thus, PBT offers a safer treatment option that can improve the patient’s quality of life during and after treatment [28]. The effectiveness of PBT is potentially limited by the capabilities of current clinical imaging methods [29]. Precise targeting is crucial for radiation planning, but standard imaging modalities like CT and standard T1-weighted contrast-enhanced (T1w-CE) MRI and T2-weighted fluid-attenuated inversion recovery (T2-FLAIR) MRI lack the specificity needed to accurately define tumor margins [30]. Additionally, pHGG routinely presents without contrast enhancement and with diffuse infiltration and ill-defined margins, making it difficult to effectively use T1w-CE and T2-FLAIR.

Whole-brain 3D spectroscopic MRI (sMRI) offers improved tumor characterization by measuring metabolite levels [31,32]. Areas of metabolically active pHGG and adult HGG are characterized by elevated choline (Cho) levels due to rapidly dividing glial cells and decreased N-acetyl aspartate (NAA) levels due to neuronal dysfunction. We determined that a two-fold increase in the Cho/NAA ratio compared to normal-appearing white matter on the contralateral side of the brain (Cho/NAA ≥ 2X) can effectively detect tumor infiltration [31]. Our group demonstrated that using sMRI to guide photon therapy in adult patients with glioblastoma demonstrated a 7-month overall survival benefit compared to standard-of-care clinical imaging-guided photon therapy [33]. Additionally, whole-brain 3D sMRI can guide biopsies in non-contrast-enhanced tumors, like adult low-grade gliomas [34,35]. Our clinical results with sMRI in the adult population motivated us to incorporate sMRI-guided radiotherapy in the pediatric population. This ongoing pilot study utilizes sMRI to guide PBT in pHGG. sMRI is incorporated into the clinical workflow, enabling its use for RT planning and monitoring longitudinal treatment responses. This report will explore unique overlap and longitudinal analyses methods that are possible due to our collection of sMRI throughout the treatment protocol. Our analysis techniques represent a novel framework for integrating advanced metabolic imaging into pediatric neuro-oncology. To our knowledge, this is the first clinical trial to use spectroscopy to guide proton beam therapy in pediatric high-grade glioma, and more broadly, the first application of spectroscopy to directly guide radiotherapy in pediatric high-grade glioma. Additionally, the inclusion of longitudinal sMRI throughout the treatment course enables a unique voxel-level overlap analysis, allowing us to map dynamic changes over time. This approach provides unprecedented insight into treatment response and disease progression and lays the groundwork for precision-guided therapy in pediatric high-grade glioma.

## 2. Materials and Methods

This study was conducted at Emory University and Children’s Healthcare of Atlanta (Atlanta, GA, USA) and was approved by the institutional review board. This study was registered with the National Clinical Trials Network (NCT04908709).

### 2.1. Study Design

**Figure 1:**
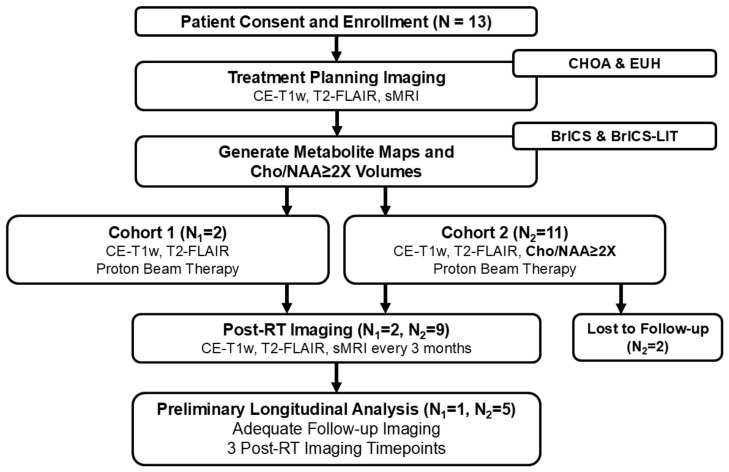
The study design. Following patient consent and enrollment by a pediatric radiation oncologist, all patients receive standard clinical imaging and whole-brain spectroscopic imaging at Children’s Healthcare of Atlanta or Emory University Hospital. The metabolic imaging is processed in BrICS and BrICS-LIT. Patients are divided into two cohorts. Targets for Cohort 1 are based solely on standard clinical imaging, and targets for Cohort 2 are based on standard clinical imaging plus the Cho/NAA ≥ 2X volume. Both cohorts receive post-RT imaging with both standard clinical imaging and sMRI every 3 months. A subset of patients with adequate follow-up timepoints was used.

Patients were recruited and enrolled if they met all eligibility criteria (see Section 2.2). See Figure 1 for the study design and number of patients enrolled. All patients underwent imaging using a consistent protocol that included standard clinical MRI (T1w-CE and T2-FLAIR) along with sMRI using a high-resolution 3D whole-brain echo-planar spectroscopic imaging (EPSI) sequence [36]. Baseline scans (sMRI and standard clinical MRI) were performed before RT to assist with treatment planning. Target treatment regions were defined using standard MRI, with high-dose radiation of 54–60 Gy guided by T1w-CE, T2-FLAIR, and elevated metabolite ratios. The specific biomarker used for RT was the region of Cho/NAA ≥ 2x, which reflected twice the average value of Cho/NAA found in normal-appearing white matter on the contralateral side of the tumor. One patient had lower signal-to-noise and scan coverage using sMRI. As a result, spectroscopy could not be used to guide radiation therapy, and this patient was assigned to Cohort 1, where proton beam therapy (PBT) was guided by standard clinical imaging.

Following the completion of six weeks of PBT, standard MRI and sMRI follow-ups commenced one month post-RT and continued every three months for up to three years. The EPSI sequence was successfully implemented and validated on the Children’s Healthcare of Atlanta (CHOA) scanner, ensuring the smooth integration of sMRI with clinical MRIs. We developed a streamlined system through the Brain Imaging Collaboration Suite (BrICS) and BrICS Longitudinal Imaging Tracker (BrICS-LIT) to enable seamless access to longitudinal sMRI data [37,38]. All baseline and follow-up clinical MRI and sMRI were reviewed prospectively by the study team, including pediatric neuroradiologists, pediatric radiation oncologists, and MR spectroscopy experts, using BrICS. This framework laid the groundwork for this study and future research requiring ongoing sMRI monitoring over time.

### 2.2. Enrollment Eligibility Criteria

To ensure consistency across all study cohorts, strict inclusion and exclusion criteria were uniformly applied. Eligible participants were required to be under the age of 21 at the time of enrollment, with a confirmed pathological diagnosis of World Health Organization (WHO) grade III–IV glioma. The primary tumor had to be in the supratentorial region of the brain, and participants had to be recommended for radiation therapy and able to undergo MRI scanning. There were no restrictions regarding gender, race, or ethnicity.

Exclusion criteria included the presence of pacemakers, non-titanium surgical clips, implants, or any non-removable medical devices incompatible with MRI. Additionally, patients were excluded if they had serious medical conditions that would impair their ability to tolerate MRI scanning or if pathology results revealed either a non-glioma diagnosis or a low-grade glioma. All participants enrolled in the study satisfied the outlined inclusion and exclusion criteria.

### 2.3. Imaging Parameters

Imaging was acquired at the CHOA Scottish Rite Hospital or Emory University Hospital. Whole-brain H-MRS EPSI parameters were modified halfway through this pilot study due to software updates to the Siemens 3T scanners and poor lipid and water suppression. Scans acquired between July 2021 and October 2023 had a scan time of 15:30 min and were acquired with GRAPPA parallelization on Siemens 3T Prisma scanners using either a 32- or 20-channel head and neck coil (echo time (TE) = 70 ms, repetition time (TR) = 975 ms, flip angle (FA) = 71°). The scan had an FOV of 170 mm × 260 mm × 120 mm and a matrix size of 64 × 50 × 22 with a nominal voxel size of 74.34 µL [36,39]. The sequence had no inversion recovery for fat suppression, allowing for a shortened TR to ensure a clinically acceptable scan time. Scans acquired after 10/2023 were acquired on the same scanner with a 32- or 20-channel head and neck coil (TE/TR/FA = 50 ms/1551 ms/71°), GRAPPA parallelization, and elliptical k-space encoding with a scan time of 15:15 min. The scan had an FOV of 280 mm × 280 mm × 180 mm and a matrix size of 50 × 50 × 18 with a nominal voxel size of 304 uL. The post-processing matrix size was 64 × 64 × 32 with an interpolated voxel size of 108 uL. Anatomic imaging with T1w and T1w-CE with a 1 mm isotropic resolution (magnetization-prepared inversion pulse, TR/TE/FA = 2300 ms/3.4 ms/9°) and T2-FLAIR with a 1 mm isotropic resolution (TR/TE/TI/FA = 7000 ms/393 ms/2100 ms/120°) were acquired during the same scan session. All raw EPSI data were processed using the Metabolite Imaging and Data Analysis System (MIDAS) (University of Miami) to obtain metabolite maps [39,40].

High-dose target volumes for sMRI-guided treatment were created using heat maps to identify regions exceeding the Cho/NAA ≥ 2X threshold. These maps were aligned with structural MRI sequences using BrICS, a customized cloud-based platform designed to integrate sMRI into radiotherapy (RT) planning [38,41]. DICOM-formatted images were first transferred to a secure, HIPAA-compliant local server and then uploaded into BrICS for processing. Within BrICS, anatomical images, individual metabolite maps, and metabolite ratio maps were interpolated and aligned to a unified coordinate system based on the isotropic high-resolution T1-weighted MRI. Contours representing regions with Cho/NAA ≥ 2x abnormalities were generated directly in the platform. T1w-CE hyperintense volumes were refined to exclude any enhancement due to post-surgical effects. All segmentation volumes underwent expert review and manual adjustments by a specialist in MR spectroscopy and a pediatric neuroradiologist (K.R). Finalized segmentation data were exported from BrICS in the DICOM format and imported into the RT planning system Eclipse/Velocity AI (Varian Medical Systems) to guide treatment delivery.

### 2.4. Longitudinal Overlap Analysis

**Figure 2:**
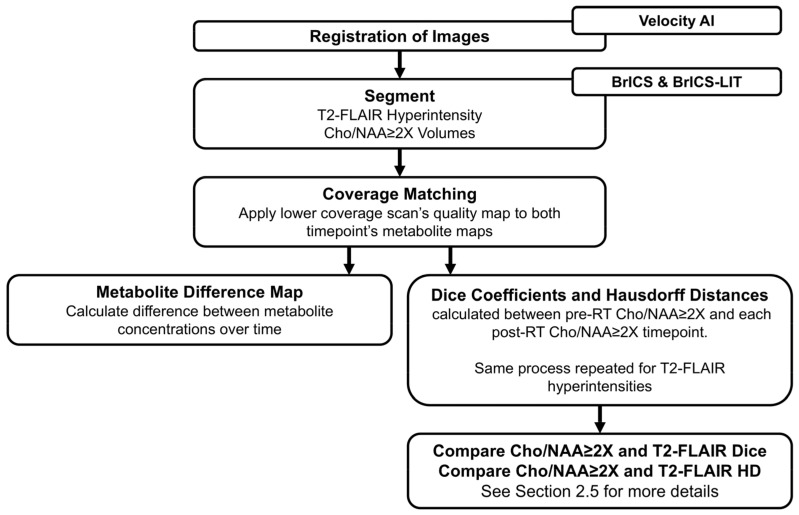
The longitudinal analysis was performed on a subset of patients collected so far in this ongoing clinical trial. The analysis was completed using pairs of timepoints. Images at each timepoint were registered to one another using Velocity AI (Varian Medical Systems). The segmentation of the T2-FLAIR hyperintensities and the Cho/NAA ≥ 2X volume was completed in BrICS and BrICS-LIT. For the metabolite maps, we performed coverage matching to ensure that both timepoints had similar metabolite coverages. Any voxels without information were excluded from both metabolite maps. Two difference analyses were performed. Differences between metabolite maps (Cho/NAA and Choline) were calculated and visualized to show voxel-level changes in metabolism over time. Overlap analyses were conducted between the pre-RT timepoint and each post-RT timepoint for the Cho/NAA ≥ 2X volume and T2-FLAIR hyperintensities.

A longitudinal analysis was conducted on a subset of patients from this ongoing clinical trial (Figure 2) [42]. The analysis involved comparing data between pairs of timepoints using only Cho/NAA ≥ 2X and T2-FLAIR volumes. T1w-CE volumes were excluded from this analysis as many of the patients did not have contrast enhancement. Images from each timepoint for each subject were aligned using Velocity AI to ensure proper registration. Pairs of follow-up scans were grouped to calculate the overlap between each pair. The segmentation of T2-FLAIR hyperintensities and the Cho/NAA ≥ 2x regions was carried out using the BrICS and BrICS-LIT platforms. All volumes for each patient at each scan were recorded. The percent change of the volume from the previous scans was also recorded and reported to radiation oncologists and neuroradiologists.

To create comparable metabolite maps across timepoints, coverage matching was performed, ensuring that both maps had consistent metabolite coverage. Voxels lacking data were excluded from both timepoints to maintain accuracy. Our first analysis visualized and quantified the metabolite difference over time by calculating differences between metabolite maps (Cho/NAA and Cho) at consecutive timepoints. These differences were visualized to highlight voxel-level metabolic changes. Second, we calculated the overlap using the Dice Similarity Coefficient (DSC) and Hausdorff distance (HD) between the pre-RT timepoints and each post-RT timepoint. The HD was used to measure the spatial discrepancy between the boundaries of regions. It calculates the maximum distance from a point on the boundary of one region to the closest point on the boundary of the other region, providing a measure of how far the regions are from perfect alignment. A higher HD indicates greater boundary differences, while a lower HD reflects closer alignment between the two regions. The DSC was used to quantify the overlap between volumes. The DSC ranges from 0 to 1, with 0 indicating no overlap and 1 indicating perfect agreement between the two regions.

### 2.5. Statistical Analysis

All statistical analyses were performed using Python 3.10 (SciPy). The relationship between the Cho/NAA ≥ 2X and T2-FLAIR hyper-intensity was visualized as a simple linear regression. Prior to analysis, both volume variables were inspected for implausible outliers and verified to satisfy linearity and homoscedasticity assumptions on residual plots; no transformation was required. The regression model provided the slope, y-intercept, Pearson correlation coefficient (R), two-tailed *p*-value for the non-zero slope hypothesis, and standard error of the estimate. A 95% confidence band for the mean regression line was generated from the standard error and visualized alongside the fitted line.

The DSC and HD were calculated on pre-RT Cho/NAA and each post-RT Cho/NAA volume. The same was performed with T2-FLAIR hyperintense volumes. The normality of each overlap measurement was evaluated using the Shapiro–Wilk test (alpha = 0.05). The DSC failed the normality assumption in both groups, whereas the HD was confirmed with Levene’s test. Consequently, DSC differences were evaluated with a two-tailed Wilcoxon signed-rank test, while HD differences were analyzed with a two-tailed paired t-test. The practical importance was quantified with a paired-sample Cohen’s d. The two primary hypotheses were controlled with a Holm–Bonferroni adjustment, with the significance set at 0.05.

## 3. Results

This ongoing study has enrolled 13 pHGG subjects. All patients are less than 21 years old and have pathologically confirmed grade III-IV glioma. Two patients in Cohort 2 were lost to follow-up, and five patients do not yet have adequate follow-up imaging to perform longitudinal overlap analysis. Of the six patients remaining, five had sMRI-guided PBT and one had standard of care MRI-guided PBT due to a lack of coverage in sMRI. See Figure 1 for details on the cohort numbers.

**Figure 3:**
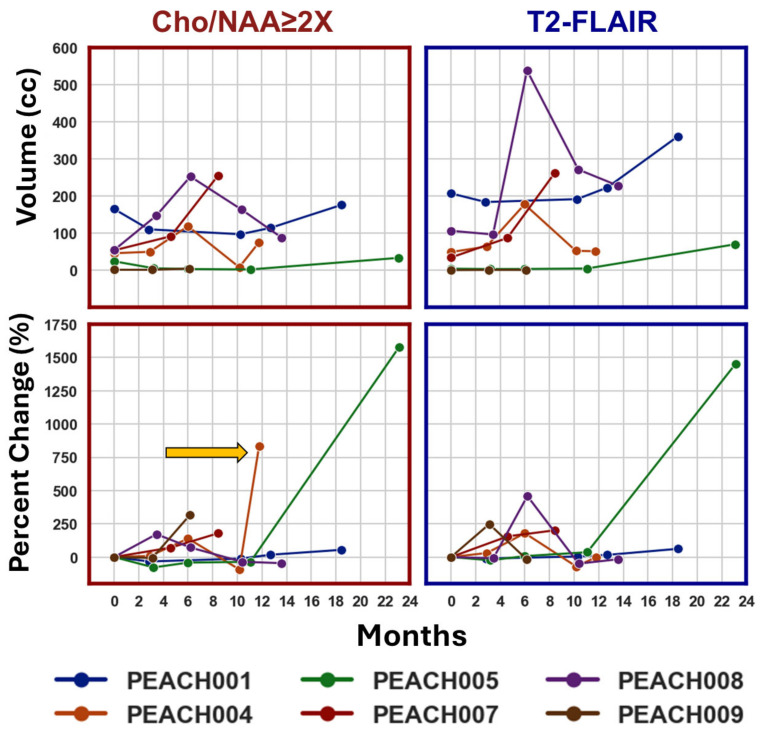
The raw volume and percent change for the Cho/NAA ≥ 2X volume (red border) and the T2-FLAIR hyperintense volume (blue border). Each patient’s raw volume over time is presented in the top row. Each patient’s percent change over time is presented in the bottom row. The yellow arrow points to a timepoint in the Cho/NAA ≥ 2X percent change, which is not represented in the T2-FLAIR percent change.

To visualize and track tumor changes over time, Figure 3 quantifies the raw volume and percent volume change of each subject as we followed them longitudinally for both Cho/NAA ≥ 2X regions and the T2-FLAIR hyperintense regions. The changes in Cho/NAA ≥ 2X preceded some volume changes found in the T2-FLAIR region.

**Figure 4:**
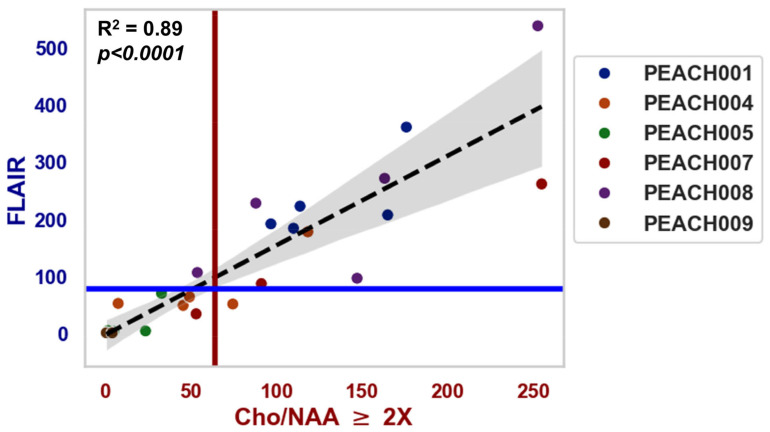
The correlation between T2-FLAIR and Cho/NAA ≥ 2X volumes. Pearson’s correlation coefficient is 0.89 (*p* < 0.0001). The red vertical line represents the median Cho/NAA ≥ 2X volume (xx cc), and the blue horizontal line represents the median FLAIR volume (cc). The dashed black line is the line of best fit, with a 95% confidence interval shown in gray.

To better visualize the relationship between spectroscopy and T2-FLAIR, Figure 4 shows the correlation between T2-FLAIR and Cho/NAA ≥ 2X volumes. There was a significant positive correlation with an R^2^ = 0.89 (*p* < 0.0001) between the two volumes when including all patients. The median FLAIR volume was 78.38 cc, and the median Cho/NAA ≥ 2X volume was 64.44 cc.

**Figure 5:**
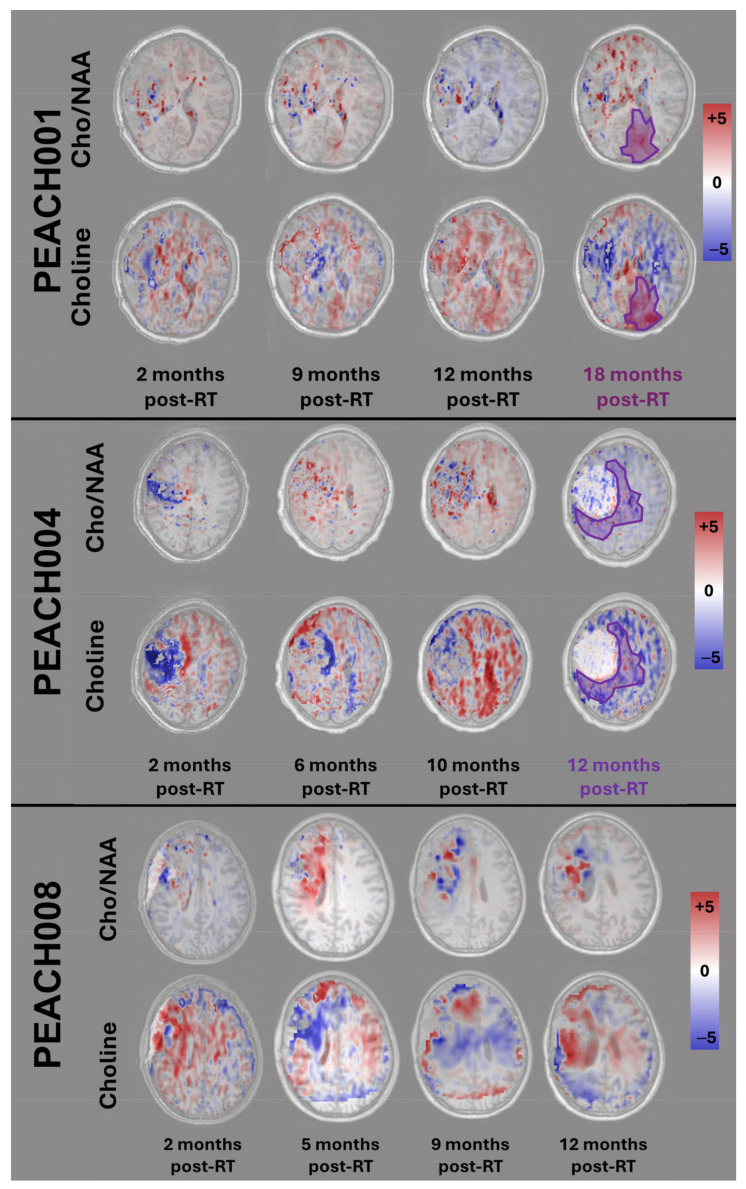
Voxel-level difference maps for representative patients. Three representative patients (PEACH001, PEACH004, and PEACH008) are shown with voxel-level changes in Choline and Cho/NAA. Progression dates and volumes are shown in purple for patients who experienced progression within this timeframe.

Complementing the volume-based analysis, Figure 5 presents the voxel-level differences in metabolic concentrations for three representative patients with adequate follow-up. The figure displays the difference between raw Cho/NAA and Cho between consecutive scans. The difference map is displayed on the T1w image from the first timepoint. Red signifies an increase in metabolite concentrations at the location, and blue signifies a decrease in metabolite concentrations in that area.

**Figure 6:**
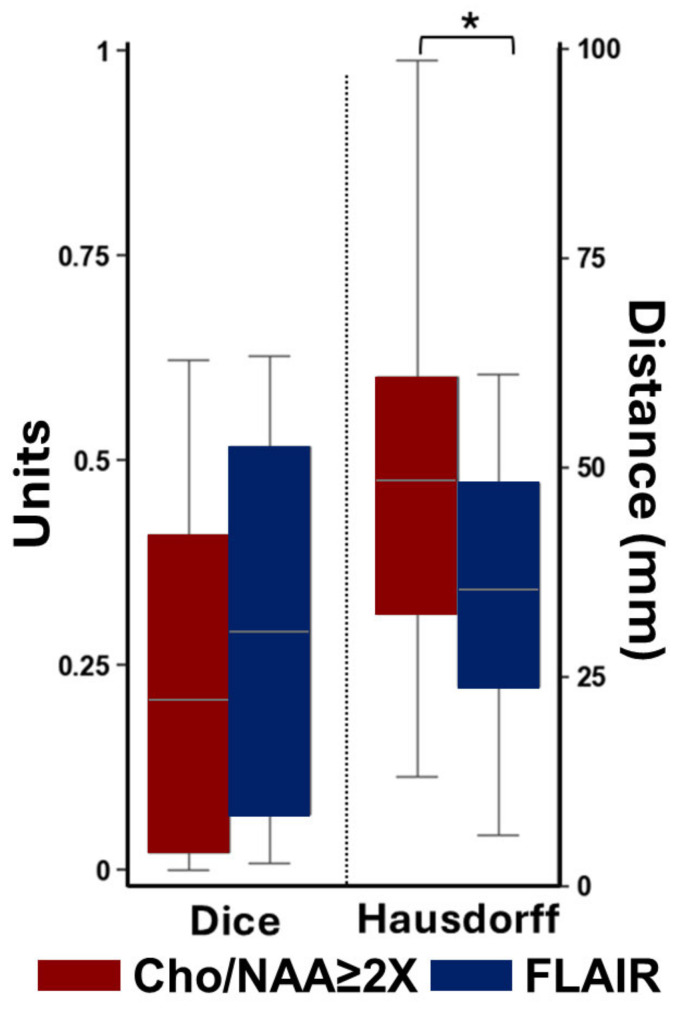
A boxplot for average overlap metrics. This shows the average DSC on the left and the Hausdorff distance on the right when we performed overlap calculations comparing pre-RT volumes to each post-RT volume. The average DSC between pre-RT Cho/NAA and post-RT Cho/NAA was lower compared to T2-FLAIR, but was not statistically significant. However, the average Hausdorff distance between pre-RT Cho/NAA and post-RT Cho/NAA was significantly higher compared T2-FLAIR metrics (* represents *p* < 0.05).

To assess the overlap between pre- and post-RT volumes, Figure 6 provides a boxplot summarizing the DSC and HD. The mean and standard deviation of the DSC between pre-RT and post-RT Cho/NAA ≥ 2X volumes was 0.24 ± 0.22 compared to T2-FLAIR DSC at 0.31 ± 0.22. DSC scores for both Cho/NAA and T2-FLAIR departed from normality (Cho/NAA W = 0.897, *p* = 0.036; FLAIR W = 0.882, *p* = 0.020). The Wilcoxon signed-rank test yielded no significant differences between Cho/NAA ≥ 2X DSC and T2-FLAIR DSC (*p* = 0.32; Cohen’s D = 0.32, small). The range of the DSC for Cho/NAA ≥ 2X was [0.00 to 0.61] and [0.01 to 0.63] for T2-FLAIR volumes. HD distances between Cho/NAA ≥ 2X (49.48 ± 20.26) and T2-FLAIR volumes (34.44 ± 16.75) were normally distributed (Cho/NAA W = 0.975, *p* = 0.862; FLAIR W = 0.959, *p* = 0.521) with homogenous variance (Levene W = 0.345, *p* = 0.561). Paired t-testing of the HD showed that there was a significant modality effect between the groups (*p* = 0.013; Cohen’s D = 0.81, large). The HD ranged from [13.04 to 98.68] for the Cho/NAA ≥ 2X comparisons to [6.08 to 61.16] for the T2-FLAIR comparisons. After Holm–Bonferroni correction (adjusted α = 0.025), the DSC comparison remained non-significant, while the HD difference persisted as statistically significant.

## 4. Discussion

This preliminary report of an ongoing pilot study shows the capabilities of longitudinal overlap analysis in the pHGG patient population. This analysis demonstrates the value of incorporating longitudinal metabolic imaging into the clinical workflow. Without acquiring sMRI every 3 months, we would have a limited understanding of the metabolic changes happening in these rapidly developing pediatric brains.

The Cho/NAA ratio maps and T2-FLAIR images in Figure 3 provide complementary insights into tumor activity and structural changes. Elevated Cho/NAA ratios reflect increased metabolic activity, indicating regions of tumor presence or progression, while T2-FLAIR hyperintensities highlight areas with edema, inflammation, or glioma infiltration. Similar changes between these two modalities suggest metabolically active regions with structural abnormalities, potentially guiding targeted treatment. However, discrepancies—where changes in Cho/NAA appear without corresponding T2-FLAIR abnormalities—may identify early tumor activity not yet visible structurally. This is seen in PEACH004’s Cho/NAA volume change at the 12-month timepoint, depicted with the yellow arrow. There is a large percent change in the Cho/NAA volume which is not represented in the T2-FLAIR graph. Retrospective review of this patient showed that progression occurred at this date. These findings underscore the importance of integrating metabolic and structural imaging for precise treatment planning, particularly in RT. In Figure 4, the positive correlation between the Cho/NAA volume and the T2-FLAIR volume suggests that areas with higher metabolic activity (elevated Cho/NAA) tend to overlap with regions showing structural disruption, such as edema or tumor infiltration. Typically, T1w-CE guides radiation oncologists towards more specific tumor targets, hence why these regions typically receive higher radiation dosages [43]. However, many pHGG patients do not display any contrast-enhancement so it is impossible to track their tumors using this metric. Thus, it is beneficial that the Cho/NAA ≥ 2X biomarkers serve as a more specific indicator of the edema and inflammation seen on T2-FLAIR and is not dependent on contrast enhancement to be visualized.

Additionally, the T2-FLAIR imaging is typically unhelpful for tumor monitoring given that inflammation from radiation can present similarly to a tumor. Figure 5 demonstrates how sMRI offers a way to differentiate true tumor progression from pseudoprogression or radiation necrosis. Typically, increases in Cho/NAA have been associated with malignancy, reflecting elevated cell membrane turnover (Cho) and reduced neuronal density or functionality (NAA) at baseline [44]. However, our results highlight that not all increases in Cho/NAA are indicative of true tumor progression in the post-treatment setting. Specifically, cases of pseudoprogression exhibit a paradoxical decrease in Cho despite an increase in the Cho/NAA ratio. This pattern may result from transient treatment effects, such as inflammation or necrosis, which decrease NAA without corresponding increases in tumor-related Cho production. In contrast, true progression is marked by concurrent increases in both Cho/NAA and Cho, consistent with tumor growth and active proliferation. This correlation between Cho/NAA and Cho suggests that increased membrane turnover remains a reliable marker for tumor progression when observed alongside a rising Cho signal [45,46,47]. This exact discrepancy can be spotted in Figure 5, PEACH008, when examining the 5-month post-RT date and the 12-month post-RT date. At 5 months, we see evidence of radiation necrosis as Cho decreases and Cho/NAA increases, and at 12 months, there is true progression around the tumor volume. These findings align with prior studies that show that elevated Cho levels are a hallmark of increased tumor burden in gliomas, emphasizing the value of Cho as a biomarker for treatment failure or recurrence [48,49,50]. Differentiating between true progression and pseudoprogression is critical, as misinterpreting pseudoprogression can lead to premature changes in therapy or unnecessary interventions. The ability to identify pseudoprogression non-invasively using sMRI biomarkers offers a significant advantage in guiding patient management. This is especially relevant in pediatric patients, where there is a critical need to accurately evaluate novel therapies for true progression, and avoiding unnecessary treatments is a priority to maximize quality of life. In addition to differentiating between true progression and pseudoprogression, metabolic alterations can precede radiographic evidence of tumor progression. For example, both PEACH001 and PEACH004 demonstrated early metabolic changes in Cho and Cho/NAA maps prior to radiologist-confirmed progression. In PEACH001, these elevations were detectable 9–12 months post-radiotherapy in regions that progressed at 18 months. PEACH004 showed increased Cho and Cho/NAA by 6–10 months post-RT, with progression confirmed at 12 months. In both cases, metabolic alterations overlapped with eventual progression, supporting the utility of sMRI as a potential early indicator of treatment failure.

Our findings also support the use of whole brain sMRI as a complement to conventional imaging modalities. Standard MRI techniques can struggle to distinguish between treatment effects and recurrent tumors, often leading to diagnostic uncertainty. The incorporation of sMRI-derived biomarkers like Cho/NAA and Cho provides a more nuanced view of tumor biology at the cellular level, improving the accuracy of progression assessments. Our analysis of spatial overlap using the DSC and the HD revealed a variability in overlap metrics and highlighted the complexity of assessing treatment response in pHGG. The DSC is a measure of overlap, while the HD measures the largest boundary mismatch between the two groups. The HD can also be interpreted as the largest distance between the edges of two volumes. Larger HDs indicate the higher differences in the margins of our volumes. We compared post-RT volumes to pre-RT volumes for both Cho/NAA and T2-FLAIR. We found that the pre- vs. post-RT Cho/NAA ≥ 2X HDs were significantly larger than pre- vs. post-RT T2-FLAIR HDs. While we noted that the DCS for this analysis was lower in pre- vs. post-RT Cho/NAA ≥ 2X compared to pre- vs. post-RT T2-FLAIR volumes, this difference was not significant. The lack of a statistically significant difference in the DSC between Cho/NAA ≥ 2X and T2-FLAIR volumes suggests that both metabolic and radiographic changes may capture aspects of the post-RT tumor environment. However, the significant difference in HDs implies that the spatial extent of metabolic disruptions may differ from radiographic abnormalities, with Cho/NAA ≥ 2X volumes often extending further. A high HD between pre- and post-RT Cho/NAA volumes reflects significant spatial change in metabolic abnormalities over time. This may indicate that regions with initially elevated Cho/NAA (e.g., tumor foci) have responded to therapy. This preliminary analysis could show the effectiveness of treating metabolically abnormal areas. However, more patients and survival information are required before we can definitively come to this conclusion. Acquiring consistent longitudinal metabolite data with sMRI allowed us to conduct these unique analyses which provide a more detailed tracking of tumor volumes throughout the treatment process. Acquiring only structural images every three months would deprive physicians of detailed quantitative information that could otherwise be gathered with sMRI. Additionally, examining differences in metabolic changes will allow physicians to visualize areas of increasing metabolic activity to better understand tumor progression and disease recurrence. To our knowledge, no prior study has serially mapped Cho/NAA at every treatment visit in pediatric high-grade glioma patients, nor related those trajectories to volumetric FLAIR response. While enrollment is still ongoing, these interim findings offer the first quantitative evidence that metabolic imaging is a valuable addition to routine structural MRI, thereby justifying broader adoption and larger confirmatory trials. This study has several limitations that should be considered when interpreting the results. First, the limited patient volume restricts the generalizability of our findings. pHGGs are relatively rare, and larger multicenter studies are needed to confirm the observed trends and validate the clinical utility of Cho/NAA and T2-FLAIR overlap metrics. Increasing the patient volume in future research will enhance the statistical power and robustness of the analysis. We are seeking to expand our study to other institutes within the Pediatric Brain Tumor Consortium to better elucidate the potential of sMRI-guided RT in pHGG patients. Second, the accuracy of our overlap metrics, including the DSC and HD, depends heavily on the quality of the spatial registration between longitudinal images. Even minor registration errors could lead to misalignment, affecting the overlap calculations. Although established registration techniques were used, these errors remain a potential source of variability and should be minimized with advanced alignment algorithms or manual corrections. Additionally, differences in resolution between sMRI and MRI may introduce partial volume effects, where a voxel contains contributions from multiple tissue types. This could impact the accuracy of Cho/NAA measurements and their alignment with T2-FLAIR hyperintensities, particularly in regions with heterogeneous pathology. The interpretation of spatial metrics also presents challenges. The DSC, while useful, is sensitive to the size of overlapping regions, and a high DSC may occur even with relatively small regions of limited clinical relevance. Similarly, the HD captures the largest boundary mismatch but may not reflect more subtle, clinically important differences within the core of the regions. We will be seeking to cross-check our overlap metrics using deep learning registration and sampling techniques. This will serve to improve our metrics’ accuracy and performance. Finally, it is important to consider that while Cho has been associated with the proliferation index (Ki-67), tumor density, and the degree of differentiation, it is not a specific biomarker [51]. Our previous histological studies confirmed that Cho/NAA is a more specific indicator of metabolically active tumors, which led us to focus on this measurement for this study. In the future, we are planning on examining the longitudinal trends of NAA in pHGG. Typically, a reduction in NAA is indicative of a decrease in the number and viability of neurons and axons. We need additional data to explore the longitudinal pattern of NAA changes in pediatric high-grade glioma. These limitations highlight the need for careful consideration when applying these metrics and underscore the importance of increasing the sample size, improving registration techniques, and incorporating additional imaging modalities to enhance the assessment of tumor response.

This preliminary report demonstrates the utility of collecting longitudinal sMRI data in pHGG. By analyzing changes in Cho/NAA ratios and Cho concentrations over time, we were able to non-invasively distinguish between true progression and pseudoprogression. The longitudinal spatial overlap analyses also showed preliminary data that suggest the benefits of using sMRI to guide RT. These findings highlight the importance of integrating advanced metabolic imaging with conventional MRI to improve diagnostic accuracy and guide clinical decision-making.

## 5. Conclusions

As the first study to integrate whole-brain spectroscopy to guide PBT in pHGG patients and track metabolic change over time, our work offers new insight into treatment response. Serial sMRI was strongly correlated with T2-FLAIR hyperintensity, yet in several cases, we showed that metabolic changes precede radiographic changes, hinting at the earlier detection of progression. Our spatial overlap metrics revealed a significantly larger HD for Cho/NAA volumes than for FLAIR volumes, indicating that the metabolic abnormalities shift more dramatically than anatomic abnormalities after therapy. Collectively, these results indicate that longitudinal whole-brain sMRI not only forecasts radiographic evolution but also uncovers spatial nuances that are invisible in structural imaging, making it a compelling tool for guiding PBT. Although the present cohort is modest and is treated at a single institution, the clear effect sizes justify multicenter validation to confirm clinical benefit and refine longitudinal endpoints for future pHGG management.

## Data Availability

The data that support the findings of this study are available on request from the corresponding author, BE.

## Abbreviations

The following abbreviations are used in this manuscript:

pHGG	Pediatric High-Grade Glioma
PBT	Proton Beam Therapy
sMRI	Spectroscopic Magnetic Resonance Imaging
EPSI	Echo-Planar Spectroscopic Imaging
Cho	Choline
NAA	N-acetyl Aspartate
BrICS	Brain Imaging Collaboration Suite
DSC	Dice Similarity Coefficient
HD	Hausdorff Distance
